# Kinase-independent role for CRAF-driving tumour radioresistance via CHK2

**DOI:** 10.1038/ncomms9154

**Published:** 2015-09-03

**Authors:** Sunil J. Advani, Maria Fernanda Camargo, Laetitia Seguin, Ainhoa Mielgo, Sudarshan Anand, Angel M. Hicks, Joseph Aguilera, Aleksandra Franovic, Sara M. Weis, David A. Cheresh

**Affiliations:** 1 Department of Radiation Medicine and Applied Sciences at the UC San Diego Moores Cancer Center, University of California, San Diego, La Jolla, California 92093, USA; 2 Department of Pathology at the UC San Diego Moores Cancer Center and Sanford Consortium for Regenerative Medicine, University of California, San Diego, 3855 Health Science Drive, La Jolla, California 92037, USA

## Abstract

Although oncology therapy regimens commonly include radiation and genotoxic drugs, tumour cells typically develop resistance to these interventions. Here we report that treatment of tumours with ionizing radiation or genotoxic drugs drives p21-activated kinase 1 (PAK1)-mediated phosphorylation of CRAF on Serine 338 (pS338) triggering a kinase-independent mechanism of DNA repair and therapeutic resistance. CRAF pS338 recruits CHK2, a cell cycle checkpoint kinase involved in DNA repair, and promotes CHK2 phosphorylation/activation to enhance the tumour cell DNA damage response. Accordingly, a phospho-mimetic mutant of CRAF (S338D) is sufficient to induce the CRAF/CHK2 association enhancing tumour radioresistance, while an allosteric CRAF inhibitor sensitizes tumour cells to ionizing radiation or genotoxic drugs. Our findings establish a role for CRAF in the DNA damage response that is independent from its canonical function as a kinase.

## Abstract (web summary)

Tumors hijack cellular pathways to evade the effects of cancer therapy. Here, Advani *et al*. show that DNA damage-induced phosphorylation of CRAF Serine 338 triggers DNA repair by recruiting CHK2, highlighting a role for CRAF independent from its canonical function as a kinase.

## Graphical abstract

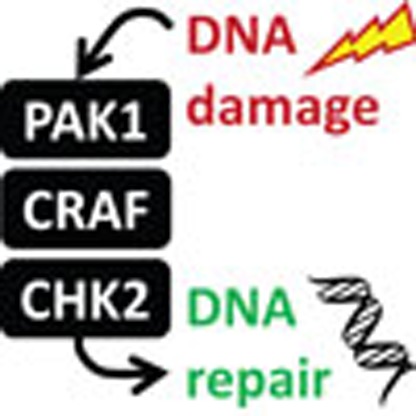

Oncogenic or stimulated RAS family GTPases are capable of triggering the growth and progression of a wide range of cancers. RAS activation drives critical downstream effectors (including PI3K, BRAF/CRAF and RAL) that not only potentiate tumour cell proliferation, and survival but also induce resistance to a wide range of therapeutics^1^,^2^,^3^,^4^. Thus, it is not surprising that targeting RAS or its effectors can sensitize tumours to the effects of genotoxic stress^5^,^6^. Consistent with the finding that CRAF becomes phosphorylated in response to ionizing radiation^7^, expression of CRAF anti-sense oligonucleotides leads to increased cellular radio-sensitivity^8^ and a liposomal formulation of the RAF anti-sense oligonucleotide LErafAON has shown promise when used in combination with radiation therapy for patients with advanced malignancies^9^. However, targeting of RAF or MEK with multi-kinase inhibitors appears to provide sensitization in some cases^10^,^11^,^12^ but not others^13^,^14^, which may be due to the non-specific nature of kinase inhibitors.

In addition to its well-known role as a kinase upstream of MEK, recent studies have uncovered a role for RAF as an adapter protein that is independent of its kinase activity^15^. CRAF phosphorylation on Serine 338 (due to P21-activated kinases) promotes CRAF association with and inactivation of the pro-apoptotic kinases ASK1 (ref. 16) and MST2 (ref. 17). CRAF forms similar complexes with ROK-α to drive cell motility^18^ and the cell cycle kinase PLK1 to drive cells through the G_2_/M cell cycle checkpoint^19^. To perturb these kinase-independent functions of CRAF, we developed an allosteric inhibitor of CRAF designed to stabilize its inactive conformation and block CRAF phosphorylation on S338 (ref. 20). This agent prevents CRAF coupling to PLK1, leading to cell cycle arrest in mitosis^19^. Given the relationship between cell cycle progression and DNA repair^21^, we considered whether CRAF pS338 might influence the DNA damage response.

Here we report that exposing tumours to radiation or genotoxic drugs induces a p21-activated kinase 1 (PAK1)-mediated phosphorylation of CRAF on S338, driving a complex between CRAF and CHK2 to promote DNA repair. Accordingly, inhibition of CRAF pS338 (but not its kinase activity) sensitizes tumour cells to radiation and genotoxic drugs by increasing the level of DNA damage. These results highlight an opportunity to target this resistance mechanism to sensitize tumours to the DNA-damaging effects of cancer therapy, potentially lowering the chemotherapy or radiation dose required to achieve tumour killing.

## Results

### CRAF protects cells from DNA damage

**Figure 1:**
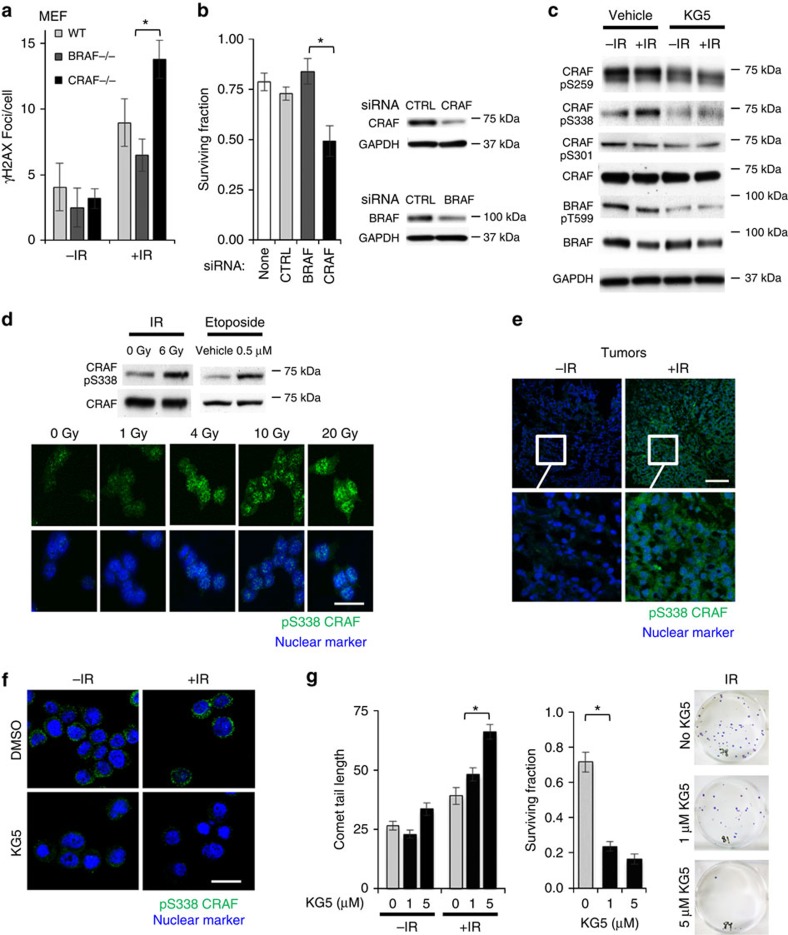
CRAF pS338 is induced by genotoxic stress and protects tumour cells from DNA damage. (**a**) Embryonic fibroblasts isolated from wild-type (WT), *BRAF*^−/−^ or *CRAF*^−/−^ mice were irradiated (6 Gy), and DNA damage was assessed using γH2AX staining. Graph shows mean γH2AX foci/cell±s.e.m. for *n*=6 fields analysed per group. ‘*’ indicates *P*<0.05 from two-sided *t*-test comparing WT and CRAF^−/−^. Data shown are representative of two independent experiments. (**b**) HCT-116 cells were transfected with siRNA to BRAF and CRAF. Cells were irradiated (2 Gy) and cell survival was measured using clonogenic survival assay. Graph shows mean surviving fraction±s.e.m. ‘*’ indicates *P*<0.05 from two-sided *t*-test comparing si-CRAF to si-BRAF, si-CTRL or non-transfected with *n*=3 wells per group. Data shown are representative of three independent experiments. (**c**) HCT-116 cells treated with KG5 (1μm) overnight, irradiated (6 Gy) and whole cell lysates collected. Immunoblotting to indicate phospho CRAF and BRAF sites. Data shown are representative of three independent experiments. (**d**) Immunoblotting for pS338 CRAF following 6 Gy or 0.5 μM etoposide. Immunostaining for CRAF pS338 (green) with dose range of IR in HCT-116 cells. Nuclei were counterstained with DAPI (blue). Scale bar, 20 μm. Data shown are representative of five fields per group for two independent experiments. (**e**) HCT-116 xenograft tumours were irradiated (6 Gy) and then immunostained for CRAF pS338 (green). Nuclei were counterstained with DAPI (blue). Scale bar, 100 μm. Data shown are representative of *n*=4 mice per group, four fields per mouse, for two independent experiments. (**f**) Immunostaining for CRAF pS338 (green) in HCT-116 cells treated with KG5 (1 μM) overnight then irradiated (6 Gy). Nuclei were counterstained with DAPI (blue). Scale bar, 20 μm. Data shown are representative of *n*=5 fields per group for two independent experiments. (**g**) HCT-116 cells were treated with KG5 and then irradiated. DNA double strand breaks were measured by neutral comet tail assay (*n*=100+ cells per group). Cell survival was measured using a clonogenic assay (*n*=3 wells per group). Bars represent mean±s.e.m. *P<0.05 from two-sided *t*-test comparing vehicle control and KG5. Data are representative of two independent experiments.

Given the well-established role for RAS activity in tumour cell resistance to therapy, we examined the relative contribution of the RAF family kinases BRAF and CRAF to radioresistance. Mouse embryonic fibroblasts isolated from *BRAF*^−/−^ or *CRAF*^−/−^ mice were exposed to radiation and monitored for DNA damage by examining the level of nuclear γH2AX foci. Only those cells deficient in CRAF showed radiosensitivity (Fig. 1a, Supplementary Fig. 1a), suggesting that CRAF but not BRAF contributed to radioresistance. We extended these studies by knocking down either CRAF or BRAF in HCT-116 human colorectal adenocarcinoma and PANC-1 human pancreatic adenocarcinoma cells and measured cell survival and DNA damage following irradiation (Fig. 1b, Supplementary Fig. 1b,c). In irradiated HCT-116 cells, knockdown of CRAF decreased cell survival. In addition, knockdown of CRAF in PANC-1 cells increased DNA damage as measured by neutral comet assay. In contrast, knockdown of BRAF in HCT-116 and PANC-1 cells had no such effect. While CRAF was required for radioresistance, MEK inhibition did not result in radiosensitization consistently (Supplementary Fig. 2). These findings suggest that, while CRAF protects cells from DNA damage, this function may not require CRAF kinase activity or MEK activation.

### DNA damage triggers phosphorylation of CRAF on Serine 338

Recent studies indicate that CRAF phosphorylated on S338 potentiates cell cycle progression, cell survival and motility in a manner that is independent of its kinase activity^15^,^16^,^18^,^19^. Given the relationship between cell cycle progression and DNA repair, we considered whether the DNA damage response might depend in part on CRAF pS338. Radiation of HCT-116 or PANC-1 cells specifically resulted in increased pS338 CRAF relative to untreated cells (Fig. 1c. Supplementary Fig. 3a). However, other phosphorylation sites on CRAF such as, pS259, pS301 or BRAF pT599 did not increase in response to radiation treatment. Furthermore, radiation treatment produced a strong dose-dependent induction of CRAF pS338 (Fig. 1d). Treatment of HCT-116 and U87 (glioblastoma) cells with the DNA-damaging cancer therapy Etoposide produced a similar CRAF pS338 dose-dependent response, suggesting that CRAF becomes phosphorylated on S338 in response to genotoxic stress (Supplementary Fig. 3b). To establish the physiological relevance of these findings, HCT-116 tumour xenografts established in mice were locally irradiated with a single dose of 6 Gy ionizing radiation (IR), harvested 2 h later, sectioned and stained for CRAF pS338. In accordance with our *in vitro* findings, tumours exposed to IR showed a marked increase in CRAF pS338 (Fig. 1e).

### CRAF pS338 is necessary and sufficient for radioresistance

We previously described a type II allosteric RAF inhibitor that stabilizes RAF in the inactivate state, known as compound 6 (ref. 20) or KG5 (ref. 19) that inhibits CRAF pS338 in various cell types. Importantly, KG5 suppressed the radiation-induced CRAF pS338 cytoplasmic staining in HCT1-116 and PANC-1 cells (Fig. 1c,f, Supplementary Fig. 4). Other phosphorylation sites on CRAF and BRAF were diminished to some degree following treatment with KG5 which is not surprising considering the fact that KG5 is an allosteric inhibitor of RAF and blocks the dimerization of BRAF and CRAF^20^ thereby preventing co-activation of these molecules. While KG5 interferes with various phosphorylation sites on RAF, only CRAF pS338 is upregulated in response to IR (Fig. 1c). Consistent with a role for CRAF pS338 in radioresistance, treatment of HCT-116 and PANC-1 cells with KG5 not only decreased clonogenic survival in response to IR, but it also markedly enhanced the DNA damage response as detected by an increased comet tail length and γH2AX foci formation (Fig. 1g, Supplementary Fig. 4).

**Figure 2:**
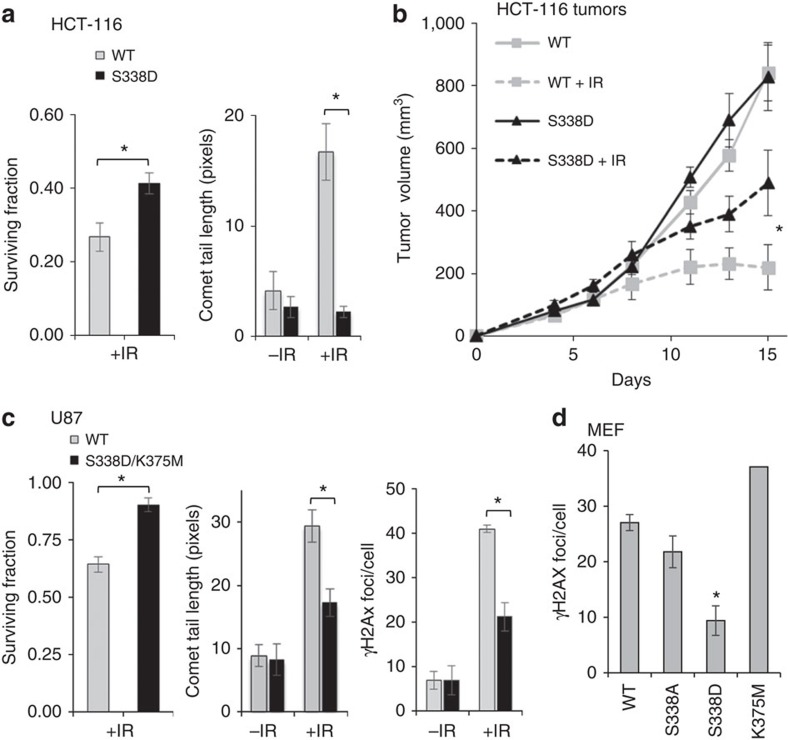
CRAF kinase activity is not required to drive radioresistance. (**a**) HCT-116 cells were stably transfected to express wild-type CRAF (WT) or the phospho-mimetic CRAF S338D mutant. Cells treated with or without 6 Gy were analysed for DNA damage using clonogenic (*P=0.012, *n*=3 wells per group, two-sided *t*-test) and comet tail assays (*P*<0.0001, *n*=100+ cells per group, two-sided *t*-test). Bars represent mean±s.e.m. Data are representative of two independent experiments. (**b**) Immune-compromised nu/nu mice were implanted s.c. with tumour cells to each thigh, and only the right thigh received three fractions of 6 Gy on Days 5, 7 and 9. Graph shows mean tumour volume±s.e.m, *P=0.04 from two-sided *t*-test comparing WT+IR (*n*=10) versus S338D+IR (*n*=9) at the endpoint on Day 15. (**c**) Stably transfected U87 cells expressing wild-type CRAF (WT) or the CRAF kinase-dead, phospho-mimetic double mutant (S338D/K375M) were exposed to 6 Gy. DNA damage was assessed by clonogenic (*P*=0.002, *n*=3 wells per group), comet tail (*P=0.0005, *n*=100+ cells per group) and γH2AX assays (*P=0.0007, *n*=6 fields per group). All bar graphs show mean±s.e.m. *P* values from two-sided *t*-tests comparing WT versus each CRAF mutant. Data are representative of two independent experiments. (**d**) *CRAF*^−/−^ MEFs were transfected with GFP-tagged WT, S338A, S338D or K375M CRAF for 72 h and then given 6 Gy. DNA damage was assessed using γH2AX staining. Graph shows mean γH2AX foci per cell±s.e.m. for *n*=50+ cells analysed per group. *P<0.05 from two-sided *t*-test comparing WT and *CRAF*^−/−^. Data are representative of three independent experiments.

To validate the role of CRAF pS338 in radioresistance, HCT-116 cells expressing a phospho-mimetic mutant of CRAF (S338D) or full-length wild-type (WT) CRAF were exposed to IR and examined for cell survival and DNA damage. Expression of CRAF S338D protected cells from IR-induced damage compared with cells expressing WT CRAF (Fig. 2a, Supplementary Fig. 5), suggesting that CRAF pS338 is sufficient to promote radioresistance. To validate this finding *in vivo*, mice were implanted with HCT-116 tumours (expressing either WT or S338D CRAF) on bilateral flanks, and only the right flank was subjected to localized 6 Gy radiation on days 5, 7 and 9 after tumour implantation. While irradiation inhibited the growth of tumours expressing WT CRAF, tumours expressing CRAF S338D continued to grow (Fig. 2b) indicating that the phospho-mimetic CRAF mutant is sufficient to protect tumours from radiation damage.

To confirm this finding and explore whether CRAF kinase activity was not required for radioprotection, U87 human glioblastoma cells expressing either WT CRAF or a double mutant phospho-mimetic/kinase-dead CRAF mutant (S338D/K375M) were subjected to IR. As previously shown, U87 cells expressing WT CRAF showed IR-induced phosphorylation of MEK, while those expressing the double mutant (S338D/375M) lacking CRAF kinase activity did not (Supplementary Fig. 6). This is consistent with the notion that this mutant CRAF is acting as a dominant negative. Importantly, cells expressing the CRAF S338D/K375M double mutant showed increased survival and reduced DNA damage following IR compared with cells expressing WT CRAF (Fig. 2c). We confirmed these results by transfecting CRAF null MEFs with WT or single mutants S338A, S338D or K375M CRAF. Null MEFs expressing WT CRAF or S338A had more DNA damage (γH2AX foci/cell) than cells expressing S338D CRAF (Fig. 2d, Supplementary Fig. 7). The K375M kinase-dead mutation produced only a slight increase in DNA damage over the control, further suggesting that S338 plays a predominant role in CRAF-mediated protection from DNA damage. These findings support the notion that CRAF pS338 is critical for DNA repair, while CRAF kinase activity is not.

### PAK1 activates CRAF pS338 and modulates radiosensitivity

**Figure 3:**
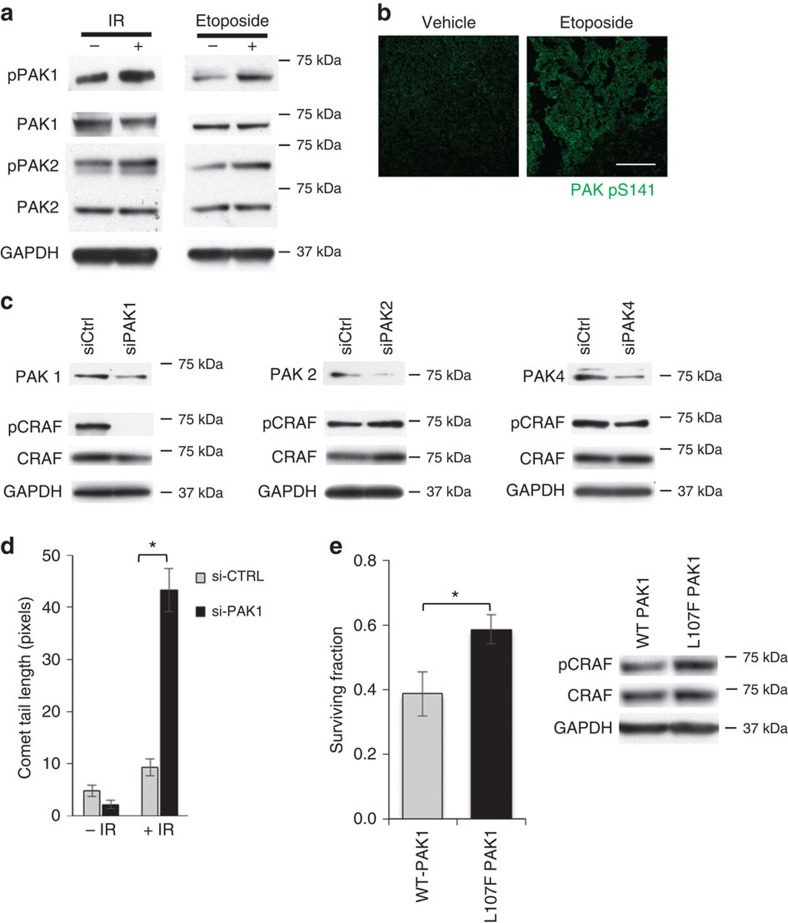
Stress-induced PAK1 triggers CRAF pS338 to prevent DNA damage. (**a**) HCT-116 cells treated with 6 Gy or 0.5 μM etoposide were lysed and analysed for total and phosphorylated PAK1/PAK2. Data are representative of three independent experiments. (**b**) HCT-116 xenograft tumours from mice exposed to Etoposide (5 mg kg^−1^) were harvested, immunostained and imaged by confocal microscopy to assess levels of PAK pS141 (green). Scale bar, 100 μm. Data shown are representative of *n*=4 mice per group, four fields per mouse, for two independent experiments. (**c**) Expression of PAK1, 2 and 4 were silenced using siRNA in HCT-116. PAK and CRAF pS338 levels were assessed by immunoblotting. Data shown are representative of two different siRNAs per target, for two independent experiments. (**d**) Expression of PAK1 was silenced using siRNA in HCT-116 cells and then cells were treated with 6 Gy. DNA damage was assessed using neutral comet assay. Mean comet tail length±s.e.m., *P<0.0001 from two-sided *t*-test comparing irradiated si-CTRL versus si-PAK1 with *n*=100+ cells per group. Data shown are representative of two different siRNAs per target, for two independent experiments. (**e**) HCT-116 cells were transfected with WT or active PAK1 (L107F) for 72 h and then irradiated. Cells were immunoblotted for pS338 CRAF and survival was measured by clonogenic assay. Mean surviving fraction±s.e.m., *P<0.05 from two-sided *t*-test comparing WT versus active PAK1 with *n*=6 wells per group.

Previous studies have shown that CRAF S338 phosphorylation depends on one or more members of the PAK family^22^,^23^,^24^,^25^. Therefore, we considered whether radiation or etoposide treatment of tumour cells would stimulate PAK activation that, in turn, would lead to CRAF pS338 and its capacity to trigger the DNA damage response. Accordingly, we found that treatment of HCT-116 cells either *in vivo* or *in vitro* with IR or etoposide resulted in enhanced activation of PAK1 and PAK2 as measured by pS141 immunoreactivity (Fig. 3a,b). Interestingly, knockdown of PAK1 (but not PAK2 or PAK4) completely abolished CRAF pS338 (Fig. 3c), and this was accompanied by a dramatic increase in IR-mediated DNA damage (Fig. 3d). Moreover, expression of constitutively active PAK1 (L017F), which increased pS338 CRAF, enhanced cell survival following radiation (Fig. 3e). Together, these findings indicate that DNA damage leads to PAK1 activation, resulting in CRAF pS338 and DNA repair.

### CRAF protects cells from DNA damage by activating CHK2

**Figure 4:**
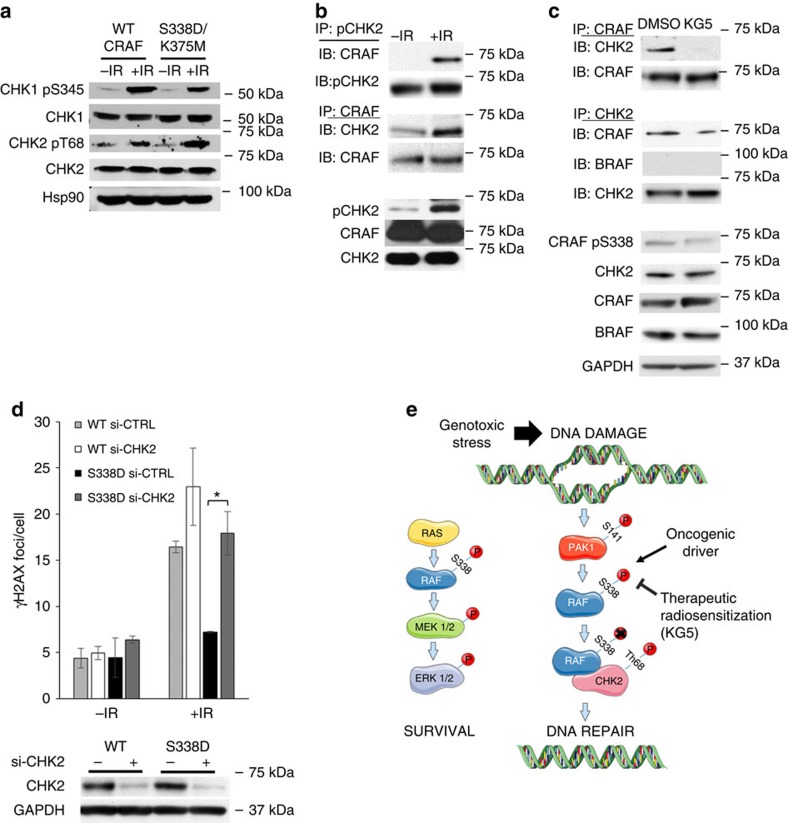
CRAF pS338 regulates the formation of a complex between CRAF and the DNA repair kinase CHK2. (**a**) Stably transfected U87 cells expressing wild-type CRAF (WT) or the CRAF kinase-dead, phospho-mimetic double mutant (S338D/K375M) were exposed to 6 Gy and immunoblotted with indicated antibodies. Data are representative of three independent experiments. (**b**) HCT-116 cells were exposed to 6 Gy. Lysates were immunoprecipitated for CHK2 pT68 and blotted for CRAF. For reciprocal pull down, lysates were immunoprecipitated and CRAF blotted for CHK2. Total cell lysates were immunoblotted with indicated antibodies. Data are representative of three independent experiments. (**c**) HCT-116 cells were treated with KG5 and lysates were immunoprecipitated for CRAF or CHK2 and immunoblotted with indicated antibodies. Total cell lysates were immunoblotted with indicated antibodies. Data are representative of three independent experiments. (**d**) Expression of CHK2 was silenced using siRNA in HCT-116 cells expressing wild-type (WT) CRAF or the phospho-mimetic CRAF S338D mutant. Cells were stained for γH2AX, and the number of γH2AX foci per cell was counted as a measure of DNA double strand breaks. Mean±s.e.m, *P=0.02 from two-sided *t*-test comparing S338D+si-CTRL versus S338D+si-CHK2 (*n*=3 fields each group). Data shown are representative of two different siRNAs for CHK2, for two independent experiments. (**e**) Schematic represents PAK1-mediated activation of CRAF pS338 in response to ionizing radiation and its recruitment of CHK2 leading to CHK2 activation that supports DNA repair and radioresistance.

An orchestrated series of genes regulate the response to the DNA-damaging effects of radiation^26^,^27^. To consider how CRAF pS338 might mediate radioprotection, RNA was extracted from U87 glioblastoma cells expressing WT CRAF or the K375M/S338D double mutant and analysed using a reverse transcription–PCR (RT–PCR) array involving 92 genes with known roles in response to DNA damage. Of the 92 genes examined, 6 showed a >2-fold increase in cells expressing the CRAF double mutant relative to WT. These include *ATM*, *ATR*, *BRCA1*, *BRCA2*, *RAD17* and *POLK*, all of which were validated by RT–PCR (Supplementary Fig. 8). Since the checkpoint kinases CHK1/CHK2 are activated by ATR/ATM and their downstream activity is modulated by BRCA1/BRCA2 (refs 28, 29), we examined the effect of the K375M/S338D mutation on CHK1/CHK2 activity. While the radiation-induced increase in active CHK1 (pS345) was similar for cells expressing WT CRAF or the CRAF K375M/S338D double mutant, active CHK2 (pT68) was upregulated twofold in cells expressing the CRAF double mutant (Fig. 4a). These findings suggest that activation of CHK2, a well-known contributor to radioresistance^30^,^31^,^32^,^33^, may partly account for the radio-protective effect of CRAF S338 phosphorylation.

Given that phosphorylation of S338 potentiates CRAF scaffolding function^16^,^19^, and the CRAF K375M/S338D double mutant leads to increased CHK2 T68 phosphorylation, we considered whether CRAF might interact with CHK2. While CRAF co-precipitated with CHK2 to some degree in non-treated cells, this interaction was markedly increased following cell exposure to radiation and included active pCHK2 (Fig. 4b). Pre-treating cells with KG5 served to decrease both the levels of CRAF pS338 and the CRAF/CHK2 association (Fig. 4c). CHK2 was able to associate with CRAF, but not with BRAF (Fig. 4c), consistent with our findings that BRAF expression was not required for radio-resistance (Fig. 1a,b). Furthermore, the CRAF/CHK2 association was enhanced in non-irradiated cells expressing the CRAF S338D phospho-mimetic mutation compared with cells expressing WT CRAF (Supplementary Fig. 9), suggesting that phosphorylation of CRAF on S338 contributes to its interaction with CHK2. To test whether CHK2 expression was required to promote the radio-protective effects of CRAF, we knocked down CHK2 in cells expressing either WT or S338D CRAF and exposed these cells to radiation. While CRAF S338D promoted radioprotection in control cells, knockdown of CHK2 completely reversed this effect (Fig. 4d). Together, these results support the notion that CRAF-mediated radioprotection depends on the ability of pS338 to couple to and facilitate the activation of CHK2, explaining why targeting CRAF pS338 can sensitize cells to DNA damage.

## Discussion

Accumulating evidence suggests that CRAF operates in a kinase-independent (and therefore MEK independent) manner to influence cellular behaviour by functioning as a molecular scaffold^15^,^16^,^19^. In particular, CRAF phosphorylation on S338 appears to contribute to CRAF scaffolding function^15^,^16^,^19^, leading to enhanced tumour cell proliferation^19^ and a significant decrease in the survival of breast cancer patients^34^. Consistent with this concept of a kinase-independent scaffolding function of CRAF, the DNA damage response mediated by CHK2 appears to depend on CRAF pS338 but not its kinase activity. CHK2 activity depended in part on its capacity to couple to CRAF pS338, however, CHK2 activation and DNA repair were not inhibited by a kinase-dead form of CRAF. In contrast to traditional RAF inhibitors that target its ATP-binding pocket to block MEK/ERK signalling, KG5 is an allosteric CRAF inhibitor that suppresses CRAF thereby disrupting its scaffolding function to produce a significant impact cell cycle progression^19^, and as shown here, the DNA damage response (Fig. 4e). While KG5 does not possess PK properties sufficient for clinical development, our results highlight the potential of such drugs as a new class of agents with the capacity to sensitize tumours to genotoxic therapies.

We have identified PAK1 as the upstream kinase that, in response to genotoxic stress, is activated and phosphorylates S338 on CRAF leading to DNA repair in a CHK2-dependent manner. PAKs respond to growth factor and integrin ligation, and their activity leads to remodelling of the cytoskeleton necessary for cell migration and invasion^35^,^36^. PAK family members have been reported to be overexpressed and or deregulated in cancers and have been linked to the DNA damage response pathway^37^. In fact, PAK1 phosphorylation and activation in response to IR have been linked to ATM, a key regulator of the DNA damage response, and microarray expression profiling has revealed that the primary group of genes regulated by PAK1 in response to DNA damage is involved in cell cycle progression^35^.

It is not surprising that DNA repair is intimately linked to cell cycle progression. On exposure to genotoxic stress, activation of various checkpoint proteins helps to ensures DNA will be repaired prior to resumption of the cell cycle. This is of paramount importance during mitosis, as unrepaired DNA damage would be propagated to daughter cells. Moreover, sensitivity to IR varies throughout the cell cycle with the G_2_/M phase being the most radiosensitive. These concepts are consistent with the roles we have uncovered for CRAF in coordinating cell progression by activation of PLK1 (ref. 19) and DNA damage repair via CHK2, both of which depend on pS338 and operate in a kinase-independent manner. The localization of CRAF pS338 at the spindle pole and its association with PLK1 may limit its potential for interaction with CHK2, thereby further contributing to the increased radiosensitivity of cells undergoing mitosis.

A number of studies have revealed the ‘RAF inhibitor paradox’ in which CRAF is activated and tumour growth enhanced for tumours with activating mutations in KRAS but not BRAF. It has become clear that ATP mimetic inhibitors of RAF drive dimerization of WT BRAF and CRAF, lead to phosphorylation of CRAF on serine 338, potentiate the MEK/ERK pathway and thereby contribute to the observed increase in tumour growth^38^,^39^,^40^. Indeed, while genetic knockdown of CRAF enhances cancer sensitivity to IR, pharmacologically targeting CRAF with ATP mimetic inhibitors often fails to radiosensitize tumours^9^,^12^,^14^. Unlike typical RAF inhibitors that target the ATP-binding pocket, the allosteric RAF inhibitor KG5 acts in a kinase-independent manner to block the dimerization of BRAF/CRAF and phosphorylation of CRAF on S338 (ref. 20). By virtue of their ability to induce cell cycle arrest in G_2_/M by targeting PLK1 (ref. 19), and to dampen the DNA repair response through the inhibition of CHK2 activity, allosteric RAF inhibitors such as KG5 would be expected to have a broad application for the sensitization of genetically diverse tumours to the effects of cancer therapies such as ionizing radiation or etoposide that function by inducing genotoxic stress.

## Methods

### Cell lines and reagents

HCT-116, PANC-1 and U87 human cancer cell lines were obtained from ATCC within the last 5 years. Cell line authentication was performed by the ATCC using short tandem repeat DNA profiles. On receipt, each cell line was expanded, cryopreserved as low-passage stocks and tested routinely for mycoplasma immediately before use in an experiment. KG5 was synthesized as previously described^20^. Sorafenib was purchased from ChemieTek, and L-779450 was purchased from Tocris. DMSO (Sigma) was used as a vehicle control for *in vitro* studies. 40% PEG400, 60% water was used as a vehicle control for *in vivo* studies, delivered as 250 μl oral gavage.

### Antibodies

All primary antibodies used for immunostaining, blotting or immunoprecipitation were purchased from commercial vendors, and were provided with datasheets that validate use with human cells. Immunoblotting was performed using the reagents and dilutions in Supplementary Table 1.

### Mutant constructs and transfection

Generation of cells stably expressing the WT CRAF, CRAF S338D and CRAF S338D/K375M constructs have previously been described^19^. BRAF and CRAF expression was transiently silenced by transfecting PANC-1 and HCT-116 cells with Qiagen BRAF (#SI02632945, #SI02632959) or CRAF(#SI0222303, #SI01396220) short interfering RNA (siRNA) constructs, CHK2 expression was transiently silenced by transfecting HCT-116 cells with a Qiagen CHK2 (#SI02655422, #SI02663857) siRNA, PAK1, PAK2 and PAK4 expression was transiently silenced by transfecting HCT-116 cells with Qiagen PAK1 (#SI00039781,#SI00039781), PAK2 (#SI00301077) and PAK4 (#SI00082341) siRNA, using lipofectamine 3000 (Invitrogen #L3000008) following manufacturer’s instructions.

### Immunofluorescent staining

Cultured cells were fixed in ice-cold methanol, permeabilized in 0.1% Triton X-100, blocked in 2% BSA, incubated with primary antibody at 1:100 dilution for 1 h at room temperature, washed and incubated in secondary antibody at 1:500 for 2 h at room temperature. Frozen tissue sections were fixed in ice-cold acetone, blocked in 0.5% BSA, incubated with primary antibody at 1:50–100 dilution overnight at 4 °C, washed and incubated in secondary antibody at 1:500 for 30 min at room temperature. Samples for γH2AX staining were exposed to 6 Gy then 2 h later were fixed in 3–4% paraformaldehyde, permeabilized in 0.25% Triton X-100, blocked in 1% BSA, incubated with primary antibody for 1 h at room temperature, washed and incubated in secondary antibody for 1 h at room temperature. Samples were incubated for 1 min in a 1:5,000 dilution of TOPRO3 or DAPI to label nuclei. Number of foci per cell was counted for 6 fields per group, with an average of 12 cells per field.

### Surviving fraction following radiation

Clonogenic assays were performed to determine the surviving fraction following exposure to 2 Gy. Briefly, cells were irradiated and then harvested 1 h later. Cells were counted and then re-plated at varying cell numbers. Colonies were counted 10–14 days after initial seeding in plates that had 20–200 colonies. For KG5-treated cells, cells were treated with 1 μM of KG5 for 16 h prior to exposure to 2 Gy.

### Comet tail assay for DNA damage

Ionizing radiation-induced DNA double strand breaks were measured using neutral comet tail assay. Cells were radiated (6 Gy), harvested 15 min after irradiation and then assayed using the Trevigen CometAssay kit. Comet tail length in pixels was measured using CometScore freeware (TriTek Corp). About 50–100 cells were analysed in each sample group.

### Immunoprecipitation and immunoblotting

Lysates were made using a standard NP-40 lysis buffer and protein concentration quantified using the Pierce BCA kit (Thermo Scientific). About 30 μg protein was loaded onto denaturing SDS–polyacrylamide gel, transferred to polyvinylidene difluoride membranes, blocked with 5% bovine serum albumin, incubated with primary antibodies overnight and HRP-conjugated secondary antibodies for 1 h (see reagent list in Supplementary Table 1), and bands detected by enhanced chemiluminescence (Pierce). Immunoprecipitation for CRAF was performed using CRAF-conjugated beads (Santa Cruz #133) or Ultralink Protein A/G Beads (VWR, #P153133) using 500 μg of protein. Uncropped scans of immunoblots are provided in Supplementary Fig. 10.

### Animal models

All animal work was approved by the UCSD Institutional Animal Use and Care Committee under protocol #S05018. Immune compromised 8–10-week-old female nu/nu mice purchased from the UCSD Animal Care Program breeding colony were injected s.c. to each thigh with five million mycoplasma-negative HCT-116 tumour cells in Matrigel (BD Biosciences). Tumour growth was measured with calipers, with volume computed as ½ × length × Width^2^. Mice were randomized into groups once the average tumour volume reached 150 mm^3^, ∼8 days after injection. Any mice with tumours±1.5 times the s.d. were excluded, as were any tumours without positive growth over the last 2 days before dosing. The remaining mice were assigned to either non-irradiated or radiated (6 Gy). Mice were killed by CO_2_ overdose followed by cervical dislocation, then dissected tumours were photographed, weighed and processed for biochemical and staining analyses.

### Ionizing radiation

The J.L. Shepherd Mark I Cesium source irradiator in the Moores UCSD Cancer Center Vivarium was used for all studies. Cells in tissue culture plates were exposed to radiation of 2 or 6 Gy. Mice with tumours are placed on a circular holder with the body shielded using a Cerrobend shield, and only the tumour region on the thigh exposed. Mice are anesthetized with ketamine/xylazine to ensure appropriate body shielding. The mice are exposed to a maximum of 6-Gy radiation, at ∼2 Gy min^−1^. These doses of radiation do not cause any constitutional symptoms since the bone marrow, lymphatic system and GI tract are not exposed.

### Experimental design and statistics

Samples were randomly assigned to groups or alternating processing order when possible, and analysis for all *in vitro* and *in vivo* experiments was performed blinded. For each assay and tumour experiment, data generated from pilot studies were used to perform power analysis to determine sample size. No samples or animals were excluded. All graphs depict mean±s.e.m. Statistical significance for all experiments was determined using a two-sided/equal variance *t*-test, with no adjustments for multiple comparisons. All samples from each group were analysed to confirm a normal distribution and equal variance. Each experiment was repeated at least twice to assure reproducibility.

## Additional information

**How to cite this article**: Advani, S. J. *et al*. Kinase-independent role for CRAF-driving tumour radioresistance via CHK2. *Nat. Commun.* 6:8154 doi: 10.1038/ncomms9154 (2015).

## Supplementary Material

Supplementary Information Supplementary Figures 1-10 and Supplementary Table 1
