# Supplementary material for: Kinase-independent role for CRAF-driving tumour radioresistance via CHK2
Source: Nat Commun. 2015 Sep 3;6:8154. doi: 10.1038/ncomms9154 (PMC4559870; doi:10.1038/ncomms9154)
Supplement: Supplementary Information — Supplementary Figures 1-10 and Supplementary Table 1 [file ncomms9154-s1.pdf]

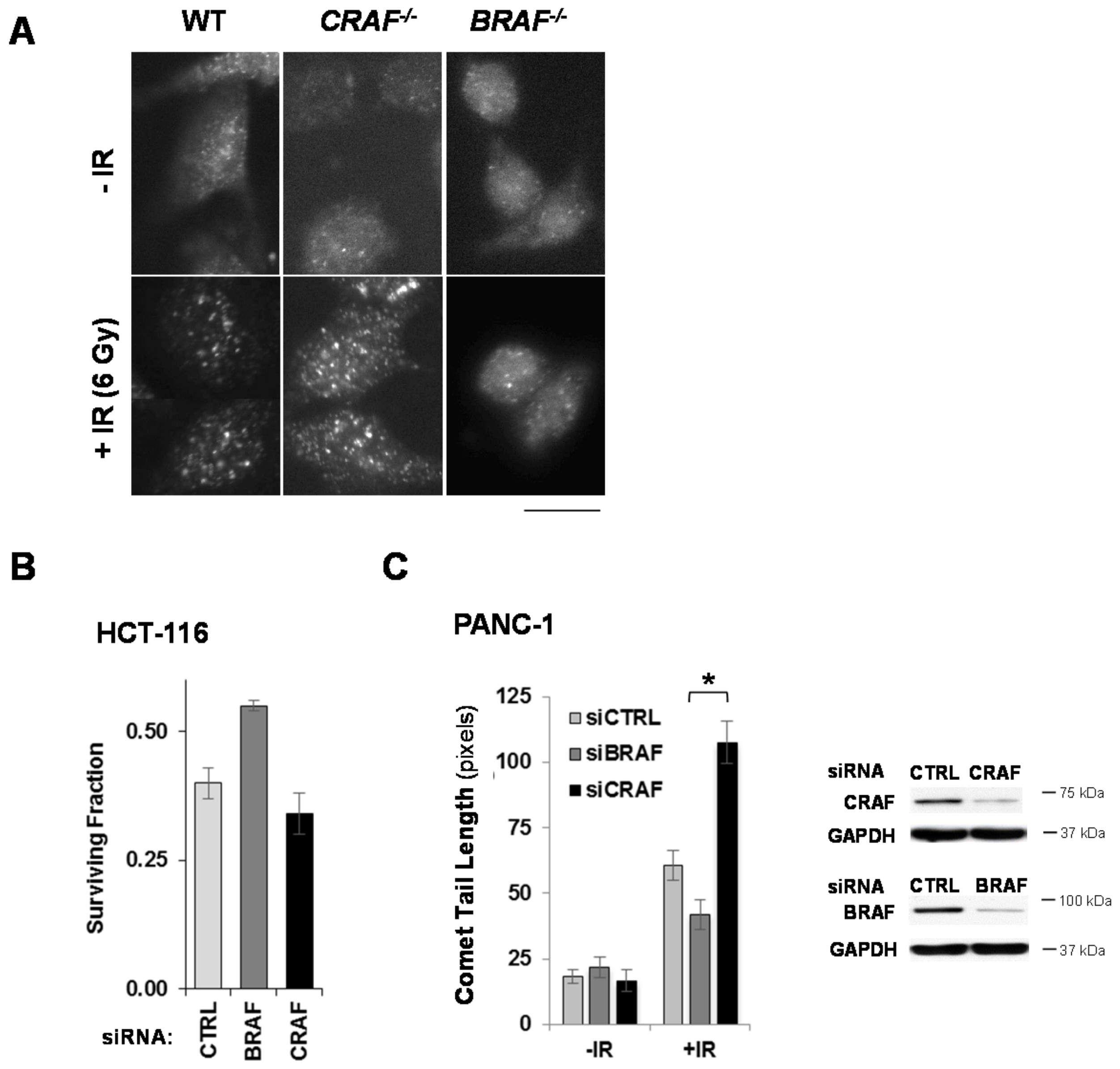

Supplementary Figure 1: si-CRAF but not si-BRAF sensitizes tumor cells to radiation.

(**a**) Embryonic fibroblasts isolated from wildtype (WT), *BRAF*$^{-/-}$, or *CRAF*$^{-/-}$ mice were irradiated (6 Gy) and DNA damage was assessed using γH2AX staining. Representative images of gH2AX foci in irradiated cells for n=6 fields per group, for two independent experiments. Scale bar = 5 μm.

(**b**) Expression of CRAF and BRAF were silenced using siRNA in HCT-116, additional siRNA than those in Figure 1b were used, cells were irradiated (2 Gy) and cell survival measured using clonogenic survival assay. Graph shows mean surviving fraction ± SEM. * indicates significant P<0.05 from 2-sided t-test comparing si-CRAF to si-BRAF or si-CTRL with n=3 wells per group. Data shown is representative of two different siRNAs per target, for two independent experiments.

(**c**) Expression of CRAF and BRAF were silenced using siRNA in PANC-1 cells, irradiated and DNA damage was assessed using neutral comet assay. Graph shows mean comet tail length ± SEM. * indicates significant P<0.0001 from 2-sided t-test comparing WT and si-CRAF with n=100+ cells per group. Immunoblot to verify siRNA knockdown of indicated proteins. Data shown is representative of two different siRNAs per target, for two independent experiments.

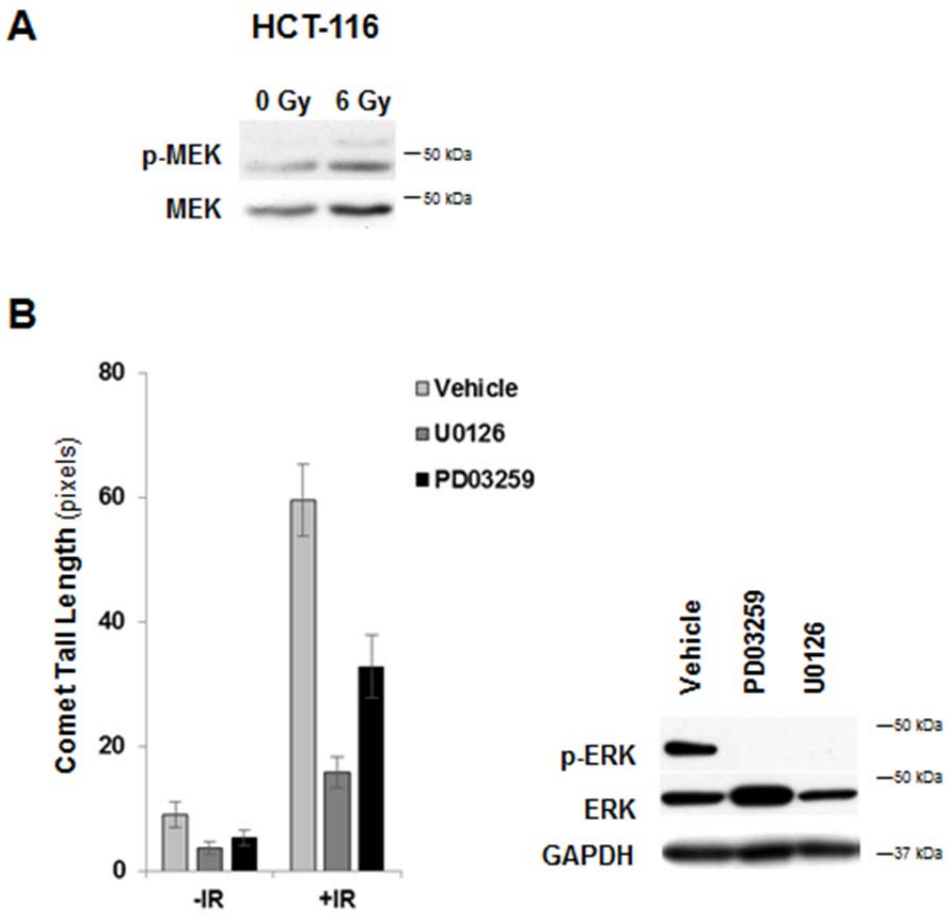

Supplementary Figure 2: Pharmacologic inhibition of MEK does not result in radiosensitization.

(**a**) Cultured HCT-116 cells were exposed to 6 Gy and then immunoblotted to assess levels of phospho-MEK. Data shown is representative of two independent experiments, and was repeated in three different cell lines.

(**b**) HCT-116 cells were treated with MEK inhibitors U0126 or PD03259, exposed to 6 Gy, and then DNA double strand breaks were measured using the comet tail assay. Graph shows mean comet tail length ± SEM for n=100+ cells per group. Immunoblot to p-ERK to confirm MEK inhibition. Data shown is representative of two independent experiments.

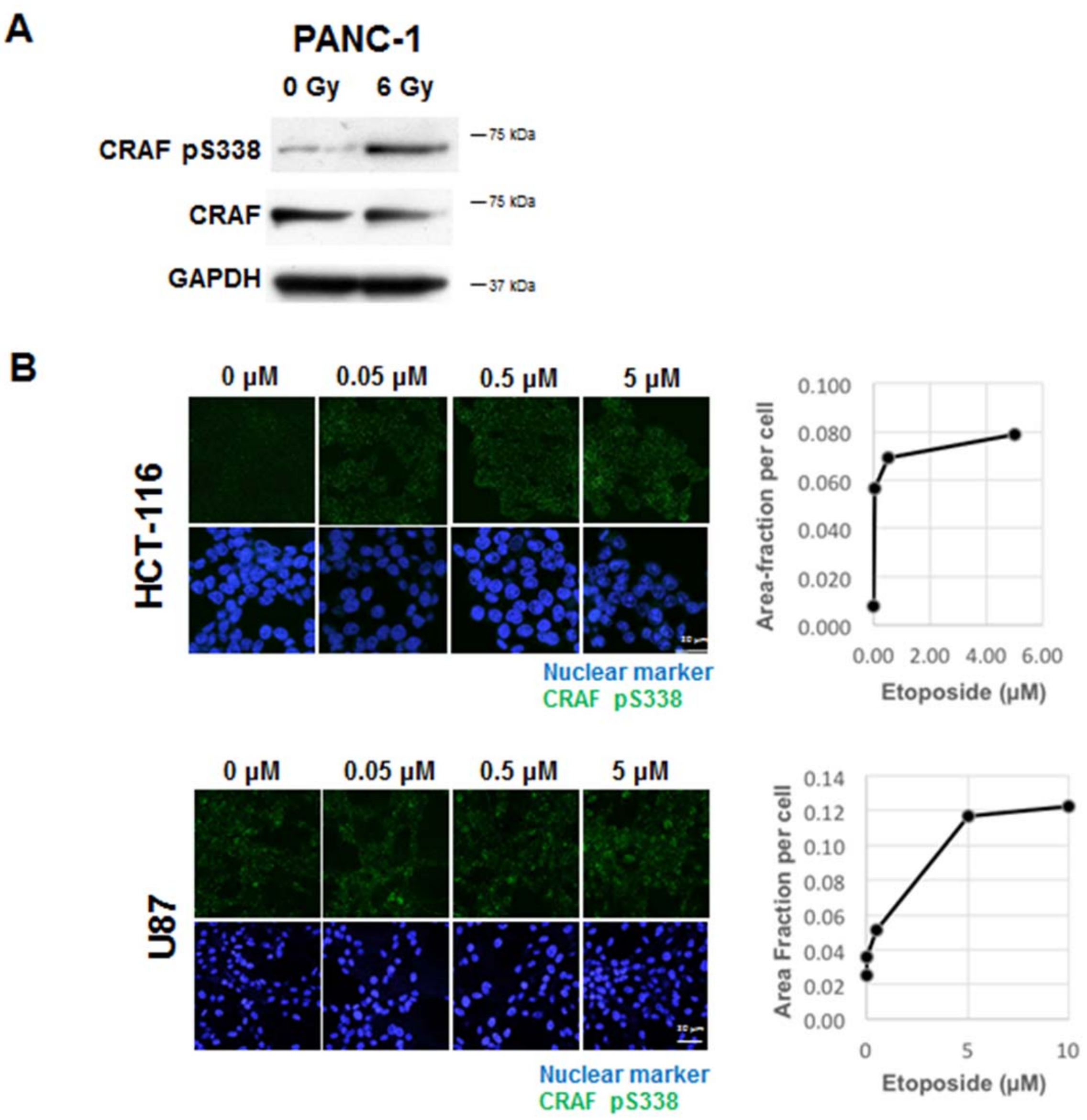

Supplementary Figure 3: DNA damage results in accumulation of pS338 CRAF.

(**a**) Immunoblotting for CRAF pS338 in irradiated PANC-1 cells. Data shown is representative of two independent experiments in two different cell lines.

(**b**) Cultured HCT-116 and U87 cells were treated with increasing concentrations of etoposide and confocal analysis to assess levels of CRAF pS338 (green). Nuclei counterstained with DAPI (blue). Graphs show mean area fraction of CRAF pS338 per cell. Data shown is representative of two independent experiments with n=5 fields per group.

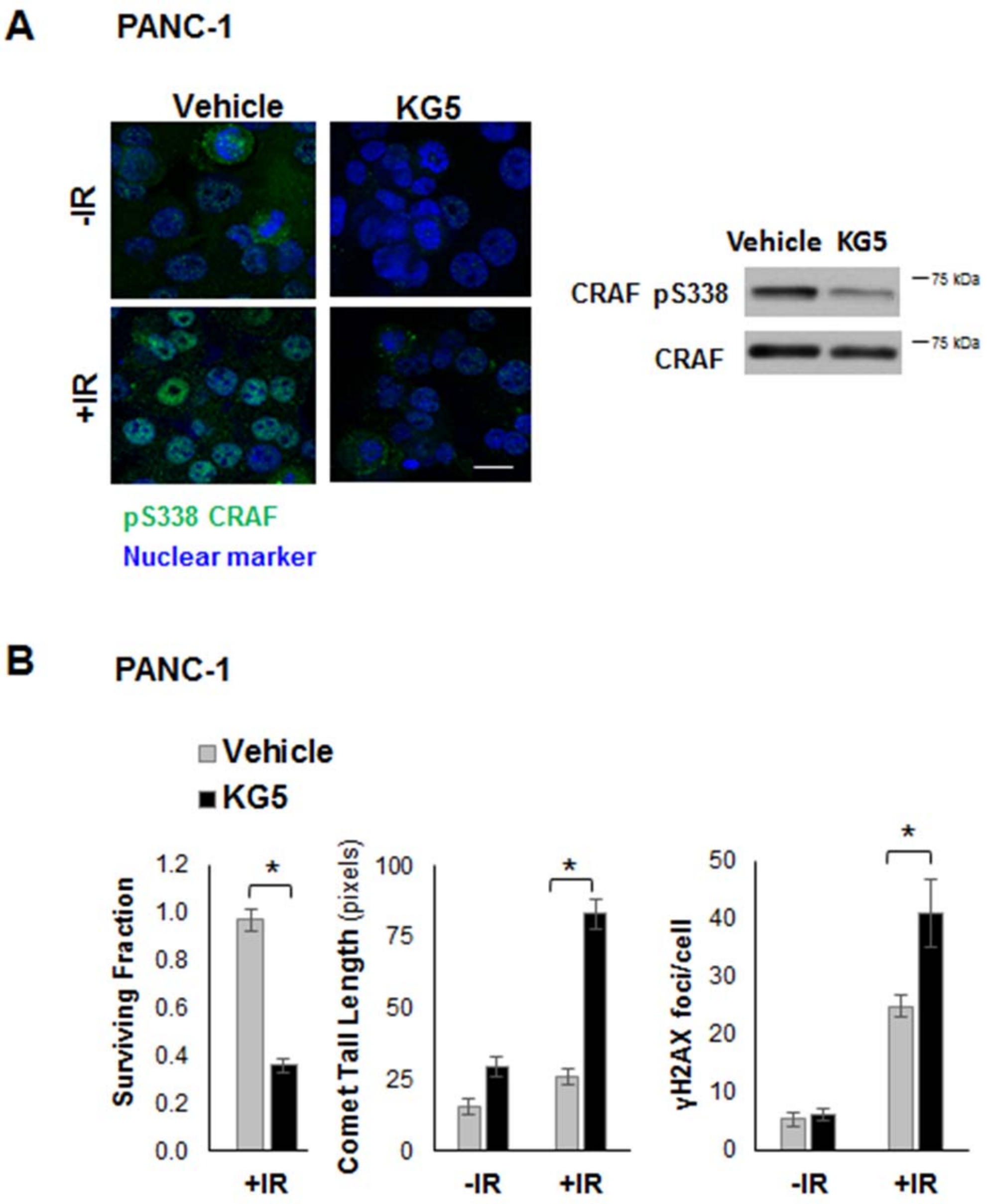

Supplementary Figure 4: KG5 inhibits pS338 CRAF and radiosensitizes PANC-1 cells.

(**a**) Immunostaining for CRAF pS338 (green) in PANC-1 cells treated with KG5 (1 μM) overnight then irradiated (6 Gy). Nuclei were counterstained with DAPI (blue). Scale bar = 10 μm. Data shown is representative of n=5 fields per group for two independent experiments.

(**b**) KG5 reduced surviving fraction (clonogenic survival assay, $P=0.004$, n=3 wells per group), DNA double strand breaks (comet tail assay, $P<0.0001$, n=100+ cells per group), and DNA damage (mean γH2AX foci per cell, $P=0.02$, n=6 fields per group). Graphs show mean ± SEM, *$P < 0.05$ vs. using 2-sided t-test comparing vehicle control and KG5. Data shown is representative of two independent experiments.

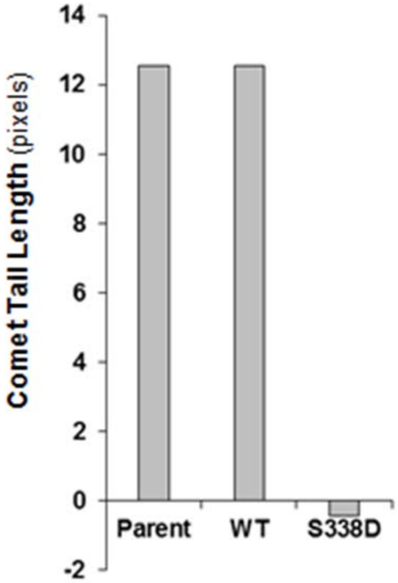

Supplementary Figure 5: S338D CRAF radioprotects compared to parental or WT CRAF expressing HCT-116 cells.

Non-transfected parental cells or stable expressing WT and S338D CRAF cells were irradiated and DNA damage measured by neutral comet assay. Comet tail following radiation for each cell line was normalized to non-irradiated cells, for n=100+ cells per group. Data is plotted mean comet tail length, representative of two independent experiments.

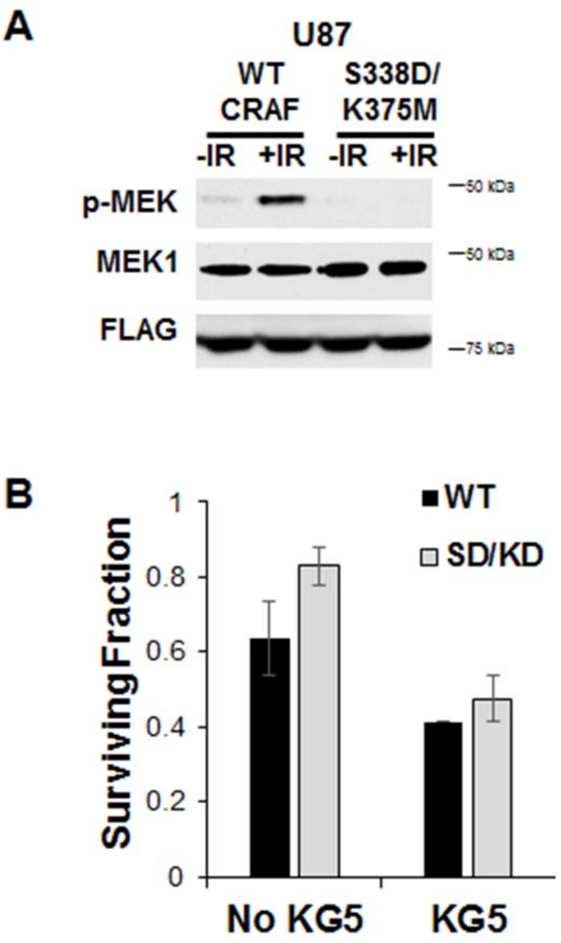

Supplementary Figure 6: S338D/K375M double CRAF mutant acts as a dominant negative.

U87 cells were transfected to express FLAG-tagged wildtype CRAF (WT) or the CRAF kinase-dead, phospho-mimetic double mutant (S338D/K375M).

(**a**) Cells were exposed to 6 Gy radiation and lysates were immunoblotted for phospho-MEK, total MEK, and FLAG. Data shown is representative of two independent experiments.

(**b**) Cells were exposed to 2 Gy radiation and DNA damage was assessed by clonogenic survival assay. Graph shows mean ± SEM for n=3 wells per group. Data shown is representative of two independent experiments.

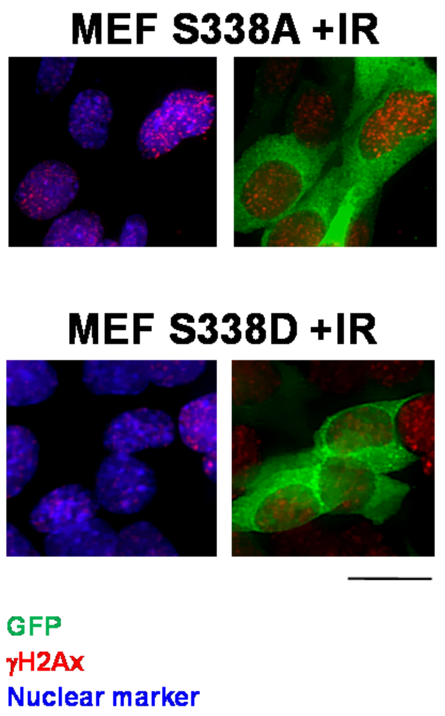

Supplementary Figure 7: CRAF -/- MEFs are radioprotected by S338D but not S338A or K375M CRAF expression.

CRAF$^{-/-}$ MEFs were transfected with GFP tagged S338A or S338D CRAF for 72 hrs and then irradiated (6 Gy). DNA damage was assessed using γH2AX staining. Representative images of 50+ cells per field for γH2AX foci in irradiated cells. GFP tagged CRAF mutant expression (green), γH2AX staining (red) and nuclei stained with DAPI (blue). Scale bar = 20 μm. Data shown is representative of three independent experiments.

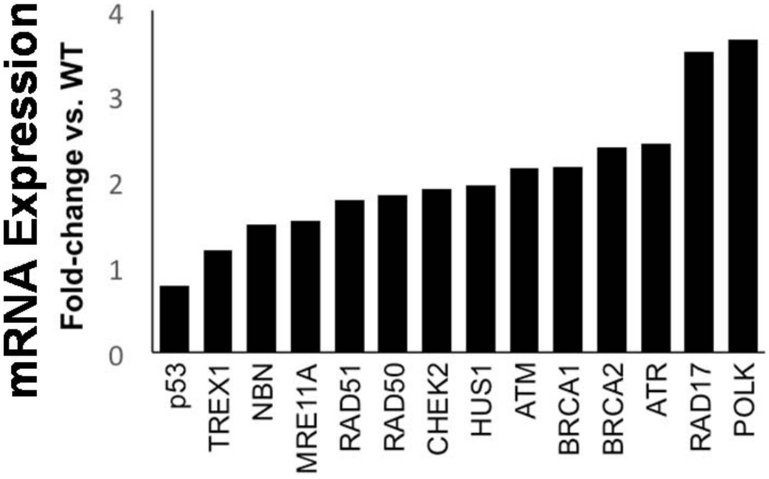

TaqMan probes	
p53	Hs01034249_m1
TREX1	Hs03989617_s1
NBN	Hs00159537_m1
MRE11A	Hs00967437_m1
RAD51	Hs00153418_m1
RAD50	Hs00990023_m1
CHEK2	Hs00200485_m1
HUS1	Hs00189595_m1
ATM	Hs01112307_m1
BRCA1	Hs01556193_m1
BRCA2	Hs00609073_m1
ATR	Hs00354807_m1
RAD17	Hs00607830_m1
POLK	Hs00211963_m1
GAPDH	Hs02758991_g1

Supplementary Figure 8: DNA damage response following IR in WT or phospho-mimetic S338 expressing cells

U87 human glioblastoma cells were transfected to express wildtype CRAF (WT) or the CRAF kinase-dead, phospho-mimetic double mutant (S338D/K375M). RNA was extracted and processed for an RT-PCR panel of genes associated with DNA damage. Changes in expression for the top 14 candidates were validated by individual RT-PCR reactions using the TaqMan probes listed (Applied Biosystems). The bar graph shows the fold-change in mRNA expression levels between U87 cells expressing WT CRAF vs. the S338D/K375M double mutant.

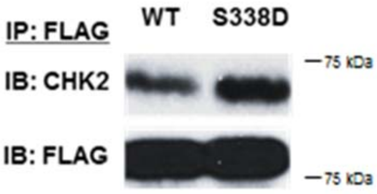

Supplementary Figure 9: Phospho-mimetic S338 CRAF has increased association with CHK2

U87 cells were transfected to express FLAG-tagged wildtype CRAF (WT) or the CRAF kinase-dead, phospho-mimetic double mutant (S338D/K375M). Lysates were immunoprecipitated for FLAG and blotted for CHK2 and FLAG. Data shown is representative of two independent experiments.

Supplementary Figure 10: Uncropped blots

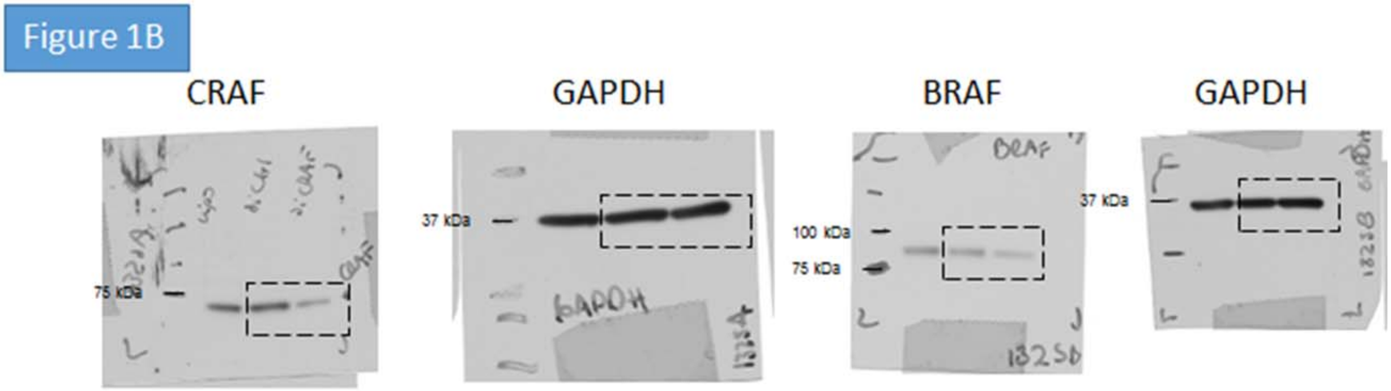

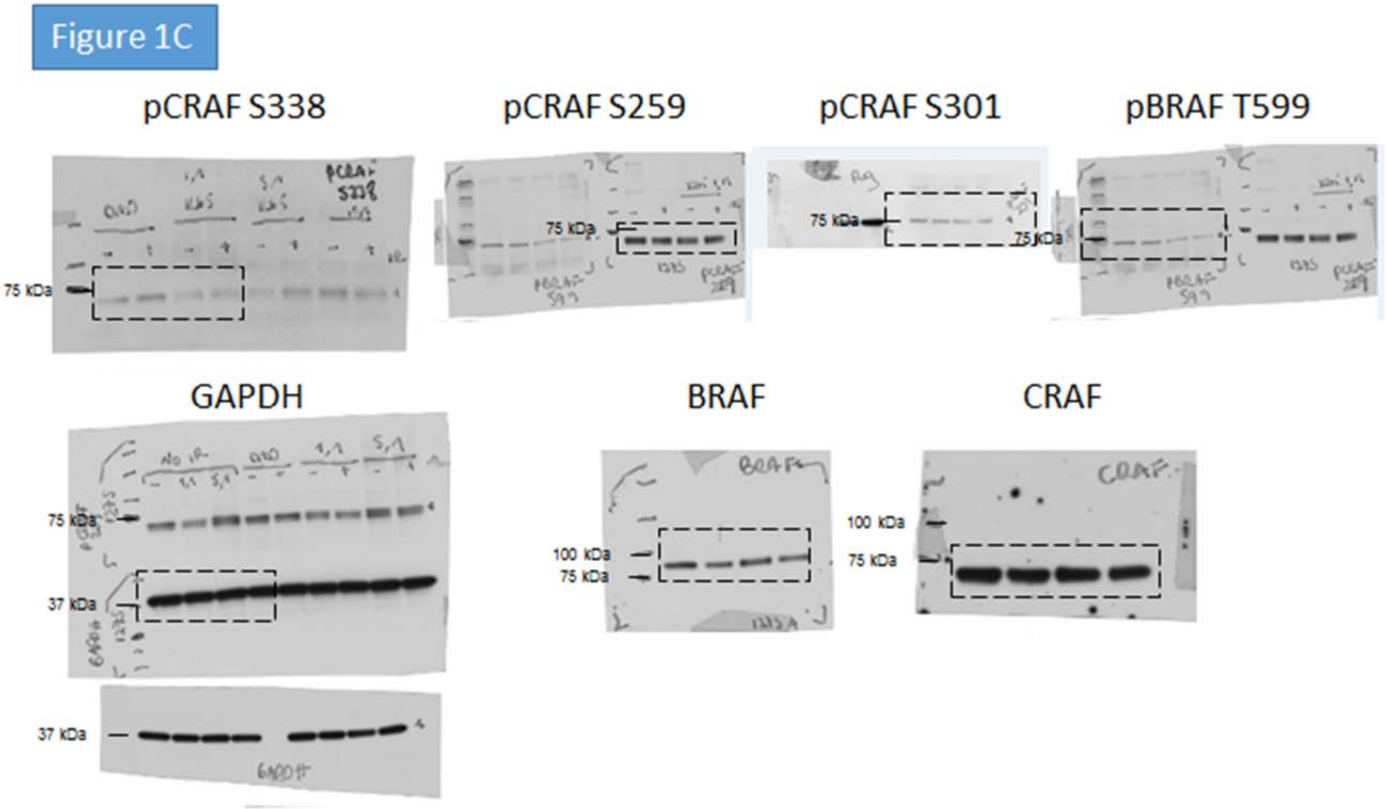

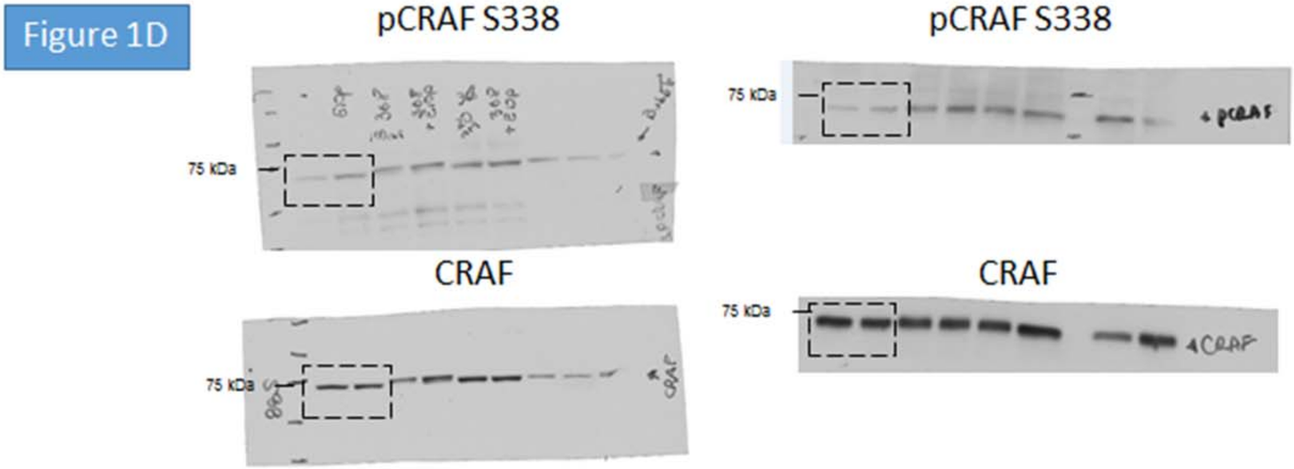

Supplementary Figure 10: Uncropped blots (continued)

Figure 3A

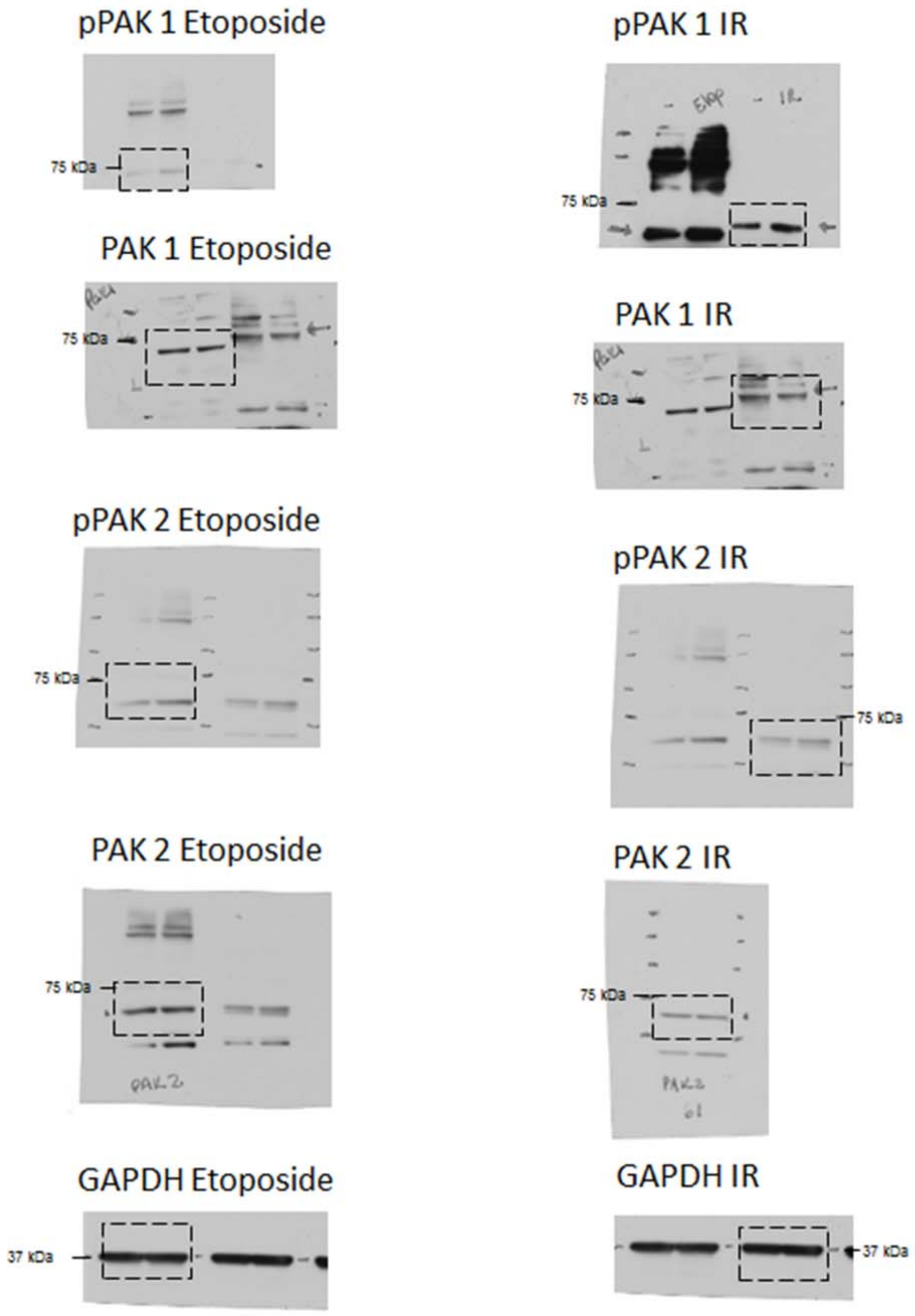

Supplementary Figure 10: Uncropped blots (continued)

Figure 3C

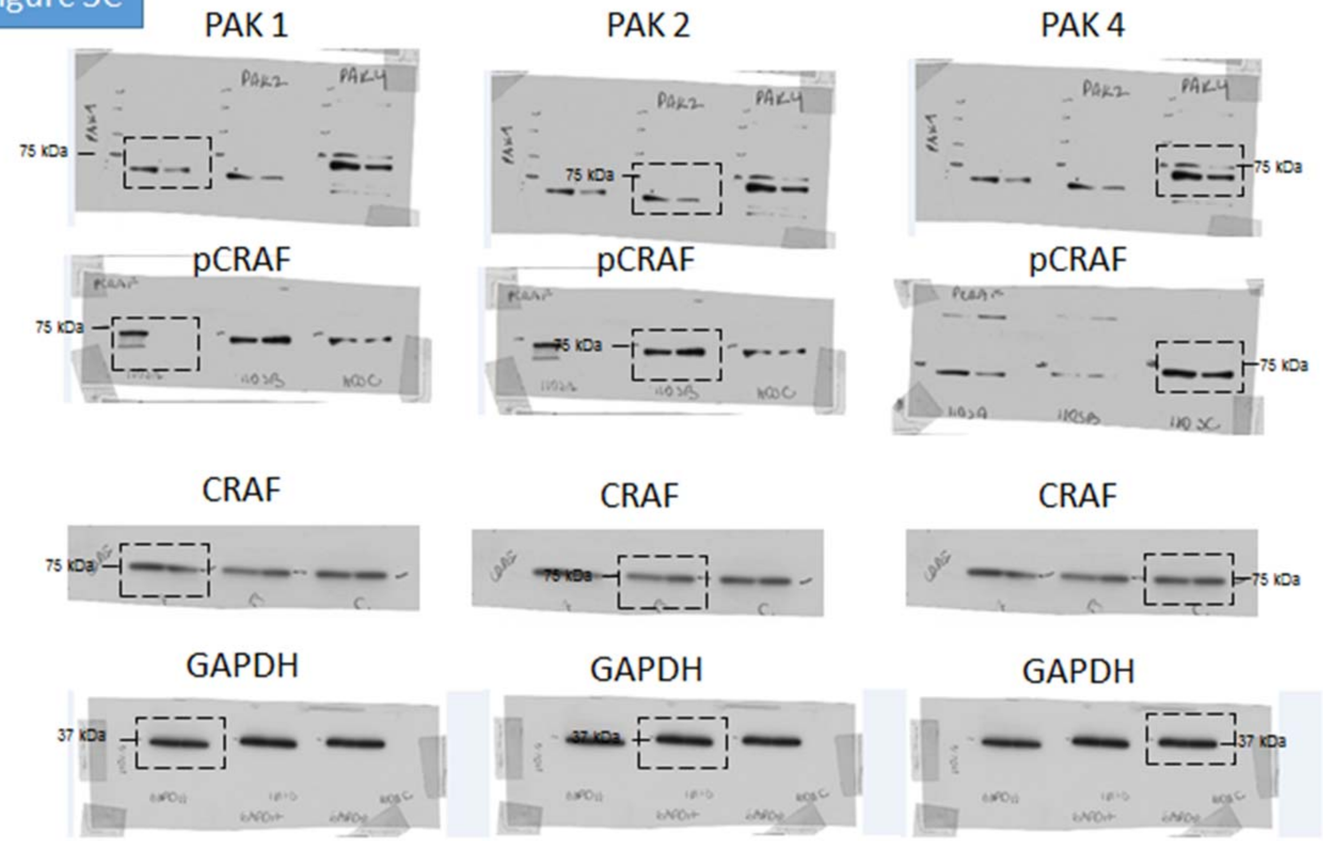

Figure 3E

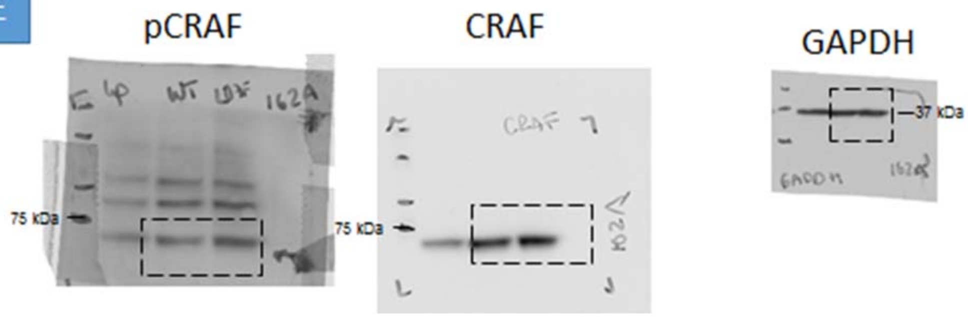

Supplementary Figure 10: Uncropped blots (continued)

Figure 4A

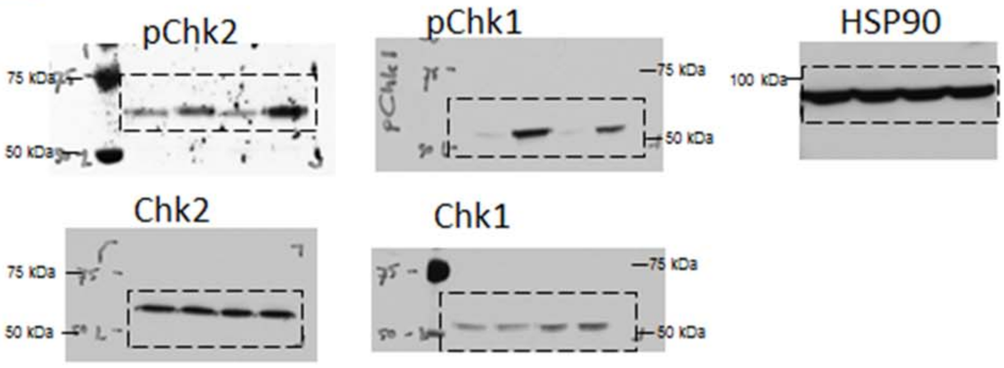

Figure 4B

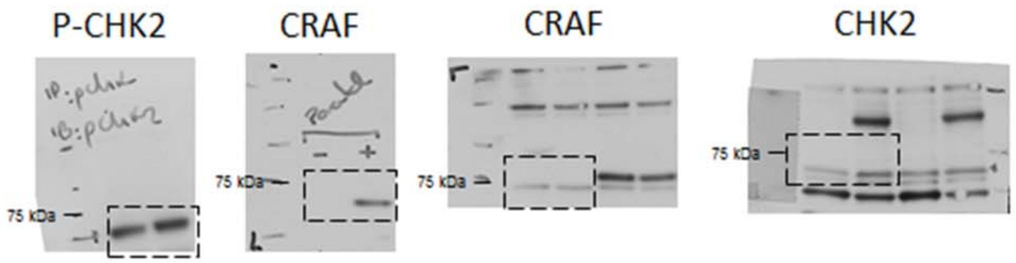

Figure 4C

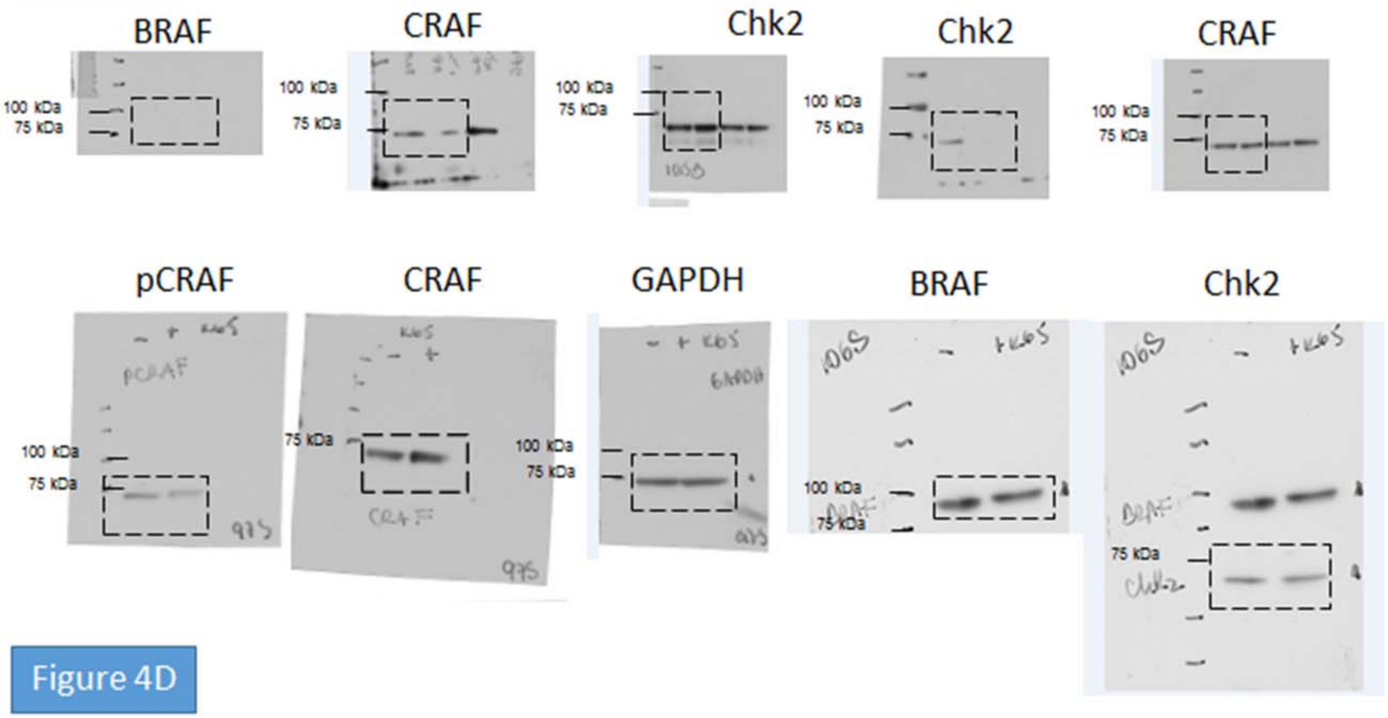

Figure 4D

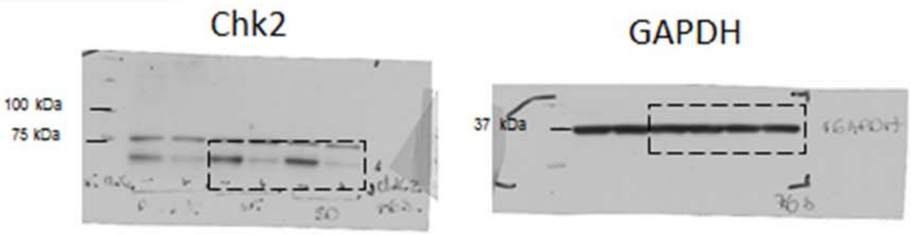

Supplementary Table 1: Antibodies for immunostaining, blotting, or immunoprecipitation

Primary Antibody	Vendor	Catalog #	Dilution
H2AX (pS139)	Abcam	11174	1:1000
FLAG	Sigma-Aldrich	F9291	1:1000
CRAF (pSer338)	Santa Cruz Biotechnology	12358	1:500
MEK (pSer217/221)	Cell Signaling	9121	1:1000
CRAF (pSer301)	Abcam	30570	1:1000
CRAF (pSer259)	Assay Biotech	A7206	1:1000
BRAF (pThr599)	Bioss Antibodies	BS-12557R	1:1000
CHK2 (pThr68)	Cell Signaling	2661	1:500
CHK1 (pSer345)	Cell Signaling	2348	1:500
CRAF	BD Transduction Labs	61015	1:1000
MEK1	Santa Cruz Biotechnology	6250	1:5000
CHK2	Cell Signaling	2662	1:1000
CHK1	Cell Signaling	2345	1:1000
PAK1	Cell Signaling	2602	1:1000
PAK2	Cell Signaling	2615	1:1000
PAK4	Cell Signaling	3242	1:1000
PAK1/2/3 (pS141)	Invitrogen	44940G	1:1000
HSP90	Santa Cruz Biotechnology	7947	1:10000
GAPDH	Cell Signaling	2118	1:10000
**Secondary Antibodies**	**Vendor**	**Catalog #**	**Dilution**
Goat Anti-Mouse IgG light chain	Jackson ImmunoResearch	115-035-174	1:1000
Goat Anti-Rabbit HRP	BioRad	170-6515	1:5000
Goat Anti-Mouse HRP	BioRad	170-6516	1:5000
**Nuclear Markers**	**Vendor**	**Catalog #**	**Dilution**
TOPRO3	Invitrogen	T3605	1:5000
DAPI	Santa Cruz Biotechnology	1306	1:5000
